# F70. COMPUTERIZED SOCIAL COGNITIVE TRAINING (SCT) IMPROVES COGNITION AND RESTORES FUNCTIONAL CONNECTIVITY IN RECENT ONSET PSYCHOSIS: AN INTERIM REPORT

**DOI:** 10.1093/schbul/sby017.601

**Published:** 2018-04-01

**Authors:** Shalaila Haas, Nikolaos Koutsouleris, Anne Ruef, Bruno Biagianti, Joseph Kambeitz, Dominic Dwyer, Ifrah Khanyaree, Rachele Sanfelici, Lana Kambeitz-Ilankovic

**Affiliations:** 1 International Max Planck Research School in Translational Psychiatry; 2 Ludwig-Maximilian-University; 3 University of California, San Francisco

## Abstract

**Background:**

Neurocognitive impairments are a core and enduring feature of psychosis that continue to persist despite pharmacological interventions. Neurocognitive interventions have emerged as a supplementary treatment option to improve cognition in early psychosis patients. Recently, focus has shifted to using social cognitive training (SCT) as evidence suggests that targeting social cognition may lead to improvements not only in cognition but also in real-world functioning (Horan et al., 2011). This improvement is thought to be mediated by restoration of functional brain activity in patients undergoing neurocognitive interventions, especially associated with medial prefrontal cortex (Hooker et al., 2014). In this study, we report our interim findings of the effects of a 10-hour SCT on cognition and resting-state functional connectivity (rsFC). Our hypothesis was that training would improve cognition and normalize functional connectivity.

**Methods:**

In this randomized-controlled study, one recent onset psychosis (ROP) patient arm (n=18) underwent a 6-week (10-hour) computerized SCT (Brain HQ, Posit Science, https://www.brainhq.com/), while another naturalistic arm (n=18) received treatment as usual (TAU). Both treatment arms were assessed on a battery of neurocognitive tests and underwent a multimodal imaging protocol, including a 10 min resting-state fMRI, at two timepoints (baseline, T0; follow-up, FU). Seed-based voxel-wise rsFC was performed and individual-level rsFC correlation maps were calculated between the bilateral medial prefrontal cortex (mPFC) and the whole brain.

**Results:**

The SCT group showed significant improvements in the domain of spatial working memory (p<0.05), processing speed (p<0.05) and resilience to both immediate and delayed memory decline over 6 weeks (p<0.05), as compared to TAU. Comparison of FC between the two measurement time points, suggested increased FC between mPFC and left inferior temporal gyrus (ITG), as well as increased FC between mPFC and left somatosensory area in ROP patients that underwent SCT relative to TAU.

**Discussion:**

We have shown improvements in processing speed, verbal memory, and spatial working memory that agree with previous studies using computerized cognitive interventions. Moreover, the improvement in the spatial working memory domain was significant in the most demanding test condition with 10 elements - indicative of benefits from the fine-tuning of higher-level executive functions. The neuroimaging results also suggested that the improvements may have been mediated by the improvement of FC in regions typically associated with social cognition and facial recognition (Adolphs et al., 2009). These results are in line with recent studies investigating not only the feasibility of SCT as an intervention, but also the effects on cognition and underlying neural alterations resulting from intensive computerized neurocognitive interventions (Hooker et al., 2012; Nahum et al., 2014; Subramaniam et al., 2014). Future studies using machine learning methods will be necessary to determine functional biomarkers in order to personalize SCT at the individual-level.

